# There are not enough data to conclude that Monomethylsilanetriol is safe

**DOI:** 10.1186/1743-7075-10-66

**Published:** 2013-10-25

**Authors:** Dirk A Vanden Berghe

**Affiliations:** 1 Department of Pharmaceutical Sciences, Faculty of Pharmaceutical, Biomedical and Veterinary Sciences, University of Antwerp, Antwerp, Belgium

**Keywords:** Monomethylsilanetriol, Orthosilicic acid

## Abstract

This article is in response to Jugdaohsingh *et al.*: The silicon supplement ‘Monomethylsilanetriol’ is safe and increases the body pool of silicon in healthy Pre-menopausal women. *Nutrition & Metabolism* 2013 10:37: http://www.nutritionandmetabolism.com/content/10/1/37

The response from the authors is published as Jugdaohsingh *et al.*: Response to Prof D. Vanden Berghe letter: ‘There are not enough data to conclude that Monomethylsilanetriol is safe’. *Nutrition & Metabolism* 2013 10:65: http://www.nutritionandmetabolism.com/content/10/1/65

**Abstract:**

The authors claim that the silicon supplement 'Monomethylsilanetriol’ (MMST) is safe and is converted to orthosilicic acid (OSA) after ingestion. Critical analysis of the study results indicates that the presented data are insufficient to conclude that the use of MMST in food or food supplements is safe. Long term safety studies in humans and toxicological testing *in vitro* and in animals are an absolute requisite for such a conclusion but these are lacking in the present study and in the literature. Furthermore, none of the presented data show that MMST is actually converted to OSA, as OSA was not analyzed in neither serum or urine of supplemented subjects.

## Correspondence/findings

Letter to the Editor

Dear Editor,

In the recent article by Jugdaohsingh et al. (Nutrition & Metabolism 2013, 10:37) the authors claim that the silicon supplement 'Monomethylsilanetriol’ (MMST) is safe and is converted to orthosilicic acid (OSA) after ingestion.

MMST has been used in liquid food supplements in Europe but no formal safety data are available. This is one of the reasons why The European Food Safety Authorithy (EFSA) [1] has given a negative opinion w.r.t. the use of MMST for nutritional purposes to food supplements and why MMST is not included in Annex II of EC Regulation n° 1170/2009 [2] which lists the vitamins and minerals and their forms that can be added to food and food supplements. Consequently, starting from January 2010, the use of MMST in food supplements is prohibited in Europe.

As background information on the biological activity of MMST, the authors refer to animal and clinical studies. The clinical studies are not placebo-controlled, double blind, randomized studies and were done in small numbers of patients. Therefore, these preliminary results should be treated with the necessary precaution and are certainly no basis to hypothesize that MMST is converted to OSA.

The authors performed a 4 weeks’ supplementation cross-over study with MMST in 22 healthy, pre-menopausal women. Since no adverse effects were reported and no biochemical changes were observed after MMST supplementation, the authors conclude that the use of MMST is safe.

But are there enough data to make such a general conclusion?

First, a supplementation period of 4 weeks is very short and therefore not sufficient, i.e. long term studies are absolutely necessary taken into account that food supplements are typically used by consumers for several months and even years.

Second, only 22 subjects were recruited of which only 14 and 18 participants had complete data sets (respectively 14 for serum and 18 for urine). It is incomprehensible how the authors make a general conclusion w.r.t. safety based on such a limited number of data. Furthermore, no reference is made to the standard battery of toxicity studies i.e. acute toxicity, subchronic and chronic toxicity, genotoxicity, reproductive and developmental toxicity studies. Have these studies been done previously? No such studies can be found on PubMed. On the contrary, in a study co-authored by one of the present authors [3], it was shown that dimethylsilanediol was highly phytotoxic and to a lesser degree also monomethylsilanetriol. Based on this documented phytoxicity it was concluded that these organic silanols cannot be considered as substitutes for silicic acid in plants. This clearly illustrates that (ortho)silicic acid is metabolized differently than organic silanols in eukaryotes. In fact, a recent study indicates that silanetriol can be seen as an analogue of silicic acid with a specific and potential therapeutic activity as it was found to inhibit the hydrolytic enzyme acetylcholinestearase [4]. In another recent study it was shown that the growth rate of the silicon dependent diatom *C. fusiformis*, was only 25–30% of the growth rate observed for silicic acid when hydroxymethylsilanetriol was used in the culture medium [5]. Analogues are frequently used in the development of new drugs but caution is certainly needed for the application in food. Profound toxicity and ADME studies are needed to evaluate the safety of these organic silanols, including MMST. MMST and similar (ortho)silicic acid analogues could also be used to elucidate the role of Si, still a *crypto* element for scientists, in connective tissue and human physiology.

In addition, I have some serious concerns regarding the study lay out.

Only young (aged 22–38 years) healthy pre-menopausal females participated the study. Men, post-menopausal women and older women were excluded. The study population is therefore not a representative sample of the normal population, which is considered as a prerequisite for performing adequate safety studies.

A minimum fasting time between the last ingestion and blood/urine sampling of 10 hours was used. However, what about the participants’ diet during these 4 weeks supplementation period? Were there any restrictions or not? Was the consumption of high Si containing sources (e.g. beans, cereals, beer) allowed and monitored? The fact that MMST was detected in some baseline urine samples (see section Discussion, pg 7) suggest that the 10-hour fasting time in this study was not sufficient and could result in a cumulative effect on silicon serum and urine concentrations. I also agree with Dr. Exley who posted a relevant comment on the Journal’s website that the authors have reported the urinary silicon excretion as mg/l for point samples which means that these results do not take into account differences in glomerular filtration rate. This implies that the reported results cannot be used to make comparisons between groups within the same study i.e. MMST supplementation versus placebo.

Nothing is mentioned about a wash-out period between the 2 treatment periods (placebo and MMST), although it is a standard procedure in cross-over studies to eliminate bias.

The analysis of MMST in serum was restricted to only 9 serum samples and 10 urine samples. After ingestion of MMST the organosilicon concentration in urine and serum was calculated to account for 10% and ca. 50% of the increase in total Si in the fasting urine and serum samples, respectively. This observation is used by the authors to conclude that MMST has been converted to OSA, without analyzing OSA itself in body fluids. I do not agree with such a conclusion for the following reasons. The authors forget to mention that after ingestion MMST can also undergo tissue loading in connective tissue and organs, which would explain why 10% is retrieved in urine and 50% in serum. Without MMST analysis in tissues and without the detection of OSA in bodyfluids one cannot conclude that MMST has been converted to OSA. The changes in silicon concentration in urine and serum, can in part be caused by differences in dietary intake and in the case of urine by differences in glomerular filtration rate, consequently calculations such as “10 or 50% of the increase in total Si” are invalid.. Interestingly, in a previous article of the authors on the bioavailability of MMST [6], they noted that the rapid absorption of MMST is “*presumably related to the organic moiety, which may allow rapid penetration of the intestinal mucosa by the molecule*”, i.e. they conclude that MMST is not converted to OSA in the GI tract. The fact that a large quantity (“50%”) of MMST is detected in serum is also contradictory to the authors’ conclusion that MMST is converted to OSA.

In the Discussion section, the authors claim that stable isotopic labeling with ^29^Si cannot be performed due to its relative high natural abundance and the relative insensitivity of ^29^Si by NMR. This is incorrect. ^29^Si NMR detection limits are sufficiently sensitive [7] as pointed out by one of the authors in a previous publication. In fact, exactly such a speciation study with ^29^Si NMR was previously done for another Si supplement, showing that this compound is converted to OSA, after ingestion [8]. This stable isotope methodology was also used by one of the authors to detect orthosilicic acid in biological samples with ^29^Si NMR [9]. Since no such speciation study was performed, concrete evidence for the conversion of MMST to OSA is totally lacking in the present study. Therefore the authors’ conclusion that following ingestion, MMST is converted to OSA is not proven by objective data.

Based on this 4 week study one cannot conclude that MMST supplementation is safe for the general public. Long-term safety studies in humans and toxicological testing *in vitro* and in animals are an absolute requisite but such studies are lacking for MMST. Furthermore, none of the presented data show that MMST is actually converted to OSA, as OSA was has not been analyzed in neither serum or urine.

## Abbreviations

MMST: Monomethylsilanetriol; OSA: Orthosilicic acid.

## Competing interests

DVB has received in the past 5 years research funds from the silicon supplement industry until September 2009.

## Authors’ contributions

DVB carried out the complete analysis and interpretation of the data and has written this publication.
